# Sucroferric oxyhydroxide decreases serum phosphorus level and fibroblast growth factor 23 and improves renal anemia in hemodialysis patients

**DOI:** 10.1186/s13104-018-3483-6

**Published:** 2018-06-08

**Authors:** Hisato Shima, Keiko Miya, Kazuyoshi Okada, Jun Minakuchi, Shu Kawashima

**Affiliations:** 1 Department of Kidney Disease, Kawashima Hospital, 1-39 Kitasakoichiban-cho, Tokushima, 770-0011 Japan; 2 Department of Internal Medicine, Kawashima Hospital, 1-39 Kitasakoichiban-cho, Tokushima, 770-0011 Japan

**Keywords:** CKD-MBD, Dialysis, FGF23, Iron-based phosphate binder, Renal anemia

## Abstract

**Objective:**

Sucroferric oxyhydroxide, a novel iron-based phosphate-binder, has been shown to have beneficial effects in lowering serum phosphorus levels and improving renal anemia in clinical studies. Although an effect of this agent on fibroblast growth factor 23 (FGF23) has been reported in an animal study, there is little clinical data supporting this finding. This study aimed to evaluate the effect on chronic kidney disease-mineral and bone disorder, FGF23, renal anemia, iron-related parameters, adverse events of sucroferric oxyhydroxide in hemodialysis patients.

**Results:**

Hemodialysis patients, receiving existing hyperphosphatemia drugs with insufficient benefit, were administered sucroferric oxyhydroxide with/without calcium carbonate for 16 weeks. Serum phosphorus level declined rapidly in Week 8 (*p* < 0.0001) and this decrease persisted until Week 16 (*p* < 0.0001). FGF23 decreased (*p* = 0.0412, Week 16), and hemoglobin increased (*p* < 0.0001, Week 16). Cumulative dose of erythropoiesis-stimulating agents (*p* = 0.0122, Week 16), and intravenous iron (*p* = 0.0233, Week 12) decreased. All adverse reactions were mild, and diarrhea was the most frequently observed adverse reaction (16.7%). Therefore, hyperphosphatemia treatment with sucroferric oxyhydroxide may safely improve serum phosphorus level, renal anemia, FGF23, and other factors that affect the prognosis of hemodialysis patients.

**Electronic supplementary material:**

The online version of this article (10.1186/s13104-018-3483-6) contains supplementary material, which is available to authorized users.

## Introduction

In patients with chronic kidney disease- mineral and bone disorder (CKD-MBD), hyperphosphatemia is reported to increase the risk of cardiovascular events resulting from vascular calcification and death [1–3], and to affect bone lesions. It has been shown that phosphate binders are able to improve hyperphosphatemia and reduce the risk of cardiovascular events and overall mortality [4, 5]. Moreover, levels of fibroblast growth factor 23 (FGF23), an endocrine hormone playing an important role in phosphorus metabolism [6], increase as CKD progresses. FGF23 is an early marker of phosphorus load [7] that has been attracting attention as an independent risk factor for cardiovascular events and mortality rate [8–10], similar to serum phosphorus levels.

Sucroferric oxyhydroxide is a novel, non-calcium-based phosphate binder with a unique complex structure consisting of polynuclear iron(III) oxyhydroxide and carbohydrate. The efficacy and safety of long-term administration have been shown in clinical studies [11–14]. Notably, sucroferric oxyhydroxide contains approximately 20% iron and has also been reported to positively affect renal anemia [15, 16]. Furthermore, in an animal study, FGF23 levels after sucroferric oxyhydroxide was administered were lower than those after lanthanum carbonate or sevelamer hydrochloride was administered [17]. However, clinical data on sucroferric oxyhydroxide in patient populations with various demographic characteristics are limited and no clinical data are available for the effects of this agent on FGF23.

Therefore, we conducted an exploratory evaluation of the clinical efficacy and safety of sucroferric oxyhydroxide given over a 16-week period in hemodialysis (HD) patients, and evaluated the effect on serum phosphorus levels, FGF23 levels and renal anemia treatment when HD patients receiving existing hyperphosphatemia drugs with insufficient benefit were switched to sucroferric oxyhydroxide.

## Main text

### Study design

This study is a prospective, one-arm, open-label study in HD patients with hyperphosphatemia. Subjects were HD (hemodialysis and hemodiafiltration) patients who visited a specialized dialysis facility in Japan (Kawashima Dialysis Clinic, Tokushima), who planned to continue their current hemodialysis regimen (≥ 3 times/week) during the study period, and who had experienced insufficient benefits, or a serum phosphorus level of > 6.0 mg/dL, while on their existing hyperphosphatemia treatment for ≥ 4 weeks before the start of this study. After the start of this study, the patient’s current hyperphosphatemia drugs, with the exception of calcium carbonate, were switched to chewable sucroferric oxyhydroxide tablets, administered orally three times a day before meals. Dose was adjusted to achieve a target serum phosphorus level of ≥ 3.5 and ≤ 6.0 mg/dL [18]. Doses of erythropoiesis-stimulating agents (ESAs) and intravenous iron (IV-iron) were also adjusted to achieve a target hemoglobin (Hb) level of ≥ 10 and < 12 g/dL [19]. For more details, please refer to the Additional file 1.

### Outcomes and assessments

CKD-MBD-related tests, Hb and iron-related tests were conducted in Weeks 0, 8, and 16 after the start of study drug administration. FGF23 and high-sensitivity C-reactive protein (hs-CRP) were measured in Weeks 0 and 16 after the start of study drug administration. The cumulative doses of ESAs and IV-iron were recorded in Week 0, 4, 8, 12, and 16.

### Statistical analysis

All data were expressed as the mean ± standard deviation (SD). Statistical analyses were performed with paired *t* test using SAS 9.4 software (SAS Institute Inc., Cary, NC, USA). *p *< 0.05 was considered to be statistically significant. Subgroup analyses were performed in two groups (switching to sucroferric oxyhydroxide and adding sucroferric oxyhydroxide to calcium carbonate monotherapy). For more details, please refer to the Additional file 1.

## Results

### Subjects

All 54 enrolled patients received sucroferric oxyhydroxide, of whom 40 patients completed 8 weeks’ treatment, and 33 patients (61.1%) completed 16 weeks’ treatment. In 21 patients, the study was discontinued due to adverse events, etc. (Additional file 2: Fig. S1). Table 1 shows patient backgrounds. Hyperphosphatemia drugs used prior to sucroferric oxyhydroxide treatment were calcium carbonate in 26 patients (65.0%) and lanthanum carbonate hydrate in 18 patients (45.0%; some patients received both agents); the mean dose of lanthanum carbonate at baseline was 1500.0 ± 500.0 mg. ESAs were used in 38 patients (95.0%), and IV-iron in 5 patients (12.5%). Six patients were newly administered calcium carbonate after the start of the study. Ten patients were administered sucroferric oxyhydroxide in addition to calcium carbonate monotherapy (Adding group). Therefore, 24 patients were switched from existing hyperphosphatemia agents to sucroferric oxyhydroxide (Switching group) (Additional file 2: Fig. S1). Characteristics of the patients in the Switching group are shown in Additional file 3: Table S1.

**Table 1:** Patient demographics and clinical characteristics

Variable	n/mean	Percentage/SD
Sex		
Male	27	67.5
Age, year	61.5	12.9
Age		
< 65 years	18	45.0
≥ 65 years	22	55.0
Dialysis method		
Hemodialysis	15	38.5
Hemodiafiltration	24	61.5
Dialysis history		
< 1 year	3	7.5
≥ 1 and < 3 years	7	17.5
≥ 3 years	30	75.0
Primary disease of dialysis		
Chronic glomerulonephritis^a^	16	40.0
Diabetic nephropathy	13	32.5
Nephrosclerosis	7	17.5
Polycystic kidney	1	2.5
Unknown	3	7.5
Complication		
Hypertension	33	82.5
Diabetes mellitus	16	40.0
Dyslipidaemia	12	30.0
Prior hyperphosphatemia drug		
Calcium carbonate	10	25.0
Calcium carbonate + lanthanum carbonate hydrate	7	17.5
Calcium carbonate + sevelamer hydrochloride	3	7.5
Calcium carbonate + bixalomer	3	7.5
Calcium carbonate + iron(III) citrate hydrate	2	5.0
Calcium carbonate + lanthanum carbonate hydrate + iron(III) citrate hydrate	1	2.5
Lanthanum carbonate hydrate	9	22.5
Lanthanum carbonate hydrate + iron(III) citrate hydrate	1	2.5
Bixalomer	4	10.0
Daily dose of lanthanum carbonate hydrate, mg	1500.0	500.0
ESAs^b^ administration	38	95.0
Cumulative dose of ESAs, IU^c^	25,315.8	18,207.7
Intravenous iron administration	5	12.5
Cumulative dose of intravenous iron, mg	160.0	0.0

The values are mean (standard deviation) or n (%). Data for the 4 weeks before the start of the study shown for ESAs and intravenous iron

^a^Including IgA nephropathy

^b^Erythropoiesis-stimulating agents

^c^International unit

### Efficacy outcomes

Serum phosphorus level had declined significantly by Week 8 (− 1.5 ± 1.6 mg/dL, *p* < 0.0001) and remained low until Week 16 (− 1.4 ± 1.8 mg/dL, *p* < 0.0001) (Table 2). Additional file 4: Fig. S2 shows the time point at which target serum phosphorus levels were achieved for the first time. The majority of patients had achieved the target for the first time by 8 weeks (70%). No significant changes were observed in adjusted calcium levels or intact- parathyroid hormone (PTH) levels (Table 2). Calcium phosphate product had decreased by Week 8 (− 13.1 ± 13.0 mg^2^/dL^2^, *p* < 0.0001) and remained low at Week 16 (− 11.7 ± 16.4 mg^2^/dL^2^, *p* = 0.0003). FGF23 had significantly decreased at Week 16 (− 4487.8 ± 12,122.0 pg/mL, *p* = 0.0412). Hb had significantly increased by Week 8 (+ 1.5 ± 1.5 g/dL, *p* < 0.0001) and remained increased at Week 16 (+ 1.7 ± 1.8 g/dL, *p* < 0.0001). The cumulative dose of ESAs had significantly decreased by Week 12 (− 8493.6 ± 14,897.5 IU, *p* = 0.0010) and was still low at Week 16 (− 9544.1 ± 20,983.4 IU, *p* = 0.0122). The cumulative dose of IV-iron had decreased by Week 12 (− 20.5 ± 54.2 mg, *p* = 0.0233) (Table 2).

**Table 2:** Efficacy parameters

	Actual value			Changes			
	n	mean	SD	n	mean	SD	p-value^m^
Serum phosphorus, mg/dL							
Baseline	40	7.0	0.7	–	–	–	–
Week 8	39	5.5	1.3	39	− 1.5	1.6	< 0.0001
Week 16	33	5.6	1.5	33	− 1.4	1.8	< 0.0001
Corrected serum calcium, mg/dL							
Baseline	40	8.6	0.9	–	–	–	–
Week 8	39	8.6	0.8	39	0.0	1.0	0.8459
Week 16	33	8.7	0.7	33	0.2	0.9	0.3332
Calcium-phosphorus products, mg^2^/dL^2^							
Baseline	40	59.8	8.4	–	–	–	–
Week 8	39	46.8	9.8	39	− 13.1	13.0	< 0.0001
Week 16	33	48.6	12.4	33	− 11.7	16.4	0.0003
Serum intact-PTH^a^, pg/mL							
Baseline	25	262.3	191.5	–	–	–	–
Week 8	19	243.6	193.9	19	− 24.0	108.5	0.3477
Week 16	22	336.1	589.3	17	− 50.4	137.7	0.1511
FGF23^b^, pg/mL							
Baseline	40	15,282.3	13,548.6	–	–	–	–
Week 16	33	10,999.7	10,594.0	33	− 4487.8	12,122.0	0.0412
Hb^c^, g/dL							
Baseline	40	11.1	1.1				
Week 8	39	12.6	1.1	39	1.5	1.5	< 0.0001
Week 16	33	12.7	1.2	33	1.7	1.8	< 0.0001
RBC^d^, × 10^−4^/μL							
Baseline	40	393.6	40.2	–	–	–	–
Week 8	39	419.1	45.7	39	25.5	36.2	< 0.0001
Week 16	33	410.5	53.0	33	18.8	53.1	0.0500
Ht^e^, %							
Baseline	40	34.9	3.1	–	–	–	–
Week 8	39	39.3	3.7	39	4.3	4.4	< 0.0001
Week 16	33	38.8	4.0	33	4.3	5.4	< 0.0001
Cumulative dose of ESAs^f^, IU							
Baseline^h^	40	24,050.0	18,594.1	–	–	–	–
Week 4^i^	38	24,236.8	20,653.1	38	− 526.3	12,019.1	0.7887
Week 8^j^	37	26,533.8	21,679.5	37	1533.8	9123.4	0.3133
Week 12^k^	39	15,762.8	15,613.6	39	− 8493.6	14,897.5	0.0010
Week 16 ^l^	34	14,985.3	15,135.9	34	− 9544.1	20,983.4	0.0122
Cumulative dose of IV-iron^g^, mg							
Baseline^h^	40	20.0	53.6	–	–	–	–
Week 4^i^	38	14.7	38.8	38	− 6.3	41.1	0.3496
Week 8^j^	37	3.2	19.7	37	− 18.4	60.1	0.0709
Week 12^k^	39	0.0	0.0	39	− 20.5	54.2	0.0233
Week 16 ^l^	34	10.6	61.7	34	− 8.2	83.4	0.5688
Daily dose of calcium carbonate, g							
Baseline	40	1.3	1.2	–	–	–	–
Week 4	38	1.3	1.2	38	0.0	0.4	0.5706
Week 8	39	1.5	1.2	39	0.2	0.6	0.0577
Week 12	40	1.4	1.2	40	0.1	0.8	0.6348
Week 16	33	1.4	1.2	33	0.1	0.9	0.3786
Daily dose of sucroferric oxyhydroxide, mg							
Baseline	40	1000.0	477.0	–	–	–	–
Week 4	38	1256.6	613.7	38	243.4	549.8	0.0097
Week 8	39	1282.1	626.1	39	269.2	591.6	0.0072
Week 12	40	1250.0	718.3	40	250.0	662.6	0.0220
Week 16	34	1323.5	727.0	34	294.1	672.7	0.0156

Cumulative doses of ESAs and intravenous iron were regarded as 0 when they were not administered

^a^Parathyroid hormone

^b^Fibroblast growth factor 23

^c^Hemoglobin

^d^red blood cell count

^e^Hematocrit

^f^Erythropoiesis-stimulating agents

^g^Intravenous iron

^h^Weeks − 4 to 0

^i^Weeks 0–4

^j^Weeks 4–8

^k^Weeks 8–12

^l^Weeks 12–16

^m^Paired *t*-test (vs. baseline)

Efficacy outcomes of the Switching group and the Adding group are shown in Additional files 5, 6, 7, 8: Tables S2–S4 and Fig. S3. In the Switching group, serum phosphorus level and calcium phosphate product had declined significantly by Week 8 and remained declined at Week 16 (− 1.4 ± 1.7 mg/dL, *p *= 0.0017; − 12.2 ± 17.9 mg^2^/dL^2^, *p *= 0.0084). Hb and Ht had significantly increased by Week 8 and remained increased at Week 16 (1.4 ± 1.5 g/dL, *p* = 0.0005; 3.6 ± 4.1%, *p* = 0.0013). The cumulative dose of ESAs had significantly decreased by Week 12 and remained decreased at Week 16 (− 12,075.0 ± 23,438.6 IU, *p *= 0.0327). The cumulative dose of IV-iron had decreased from Week 4 to Week 16 (− 32.0 ± 65.7 mg, *p* = 0.0421). No significant changes were observed in the adjusted calcium levels, intact-PTH, and FGF23 levels.

### Safety outcomes

As shown in Table 3, serum iron, serum ferritin, and transferrin saturation (TSAT) levels increased from baseline at Week 8, and serum ferritin level continued to rise through to Week 16 (90.6 ± 79.5 ng/mL). Hs-CRP levels increased from baseline at Week 16. Adverse reactions occurred in 18 of 54 patients (33.3%); the most frequent event was diarrhea, which was observed in 9 of 54 patients (16.7%) (Additional file 9: Table S5). Serious adverse events were observed in 8 of 54 patients (14.8%), none of which were found to have a causal relationship with sucroferric oxyhydroxide. In terms of the safety parameters and adverse reactions of the Switching group and the Adding group, please refer to the Additional files 10, 11: Tables S6, S7.

**Table 3:** Safety parameters

	Actual value			
	n	Mean		SD
Serum iron, μg/dL	Week 0	54	60.1	33.5
	Week 8	33	82.1	23.5
	Week 16	33	80.2	29.0
Serum ferritin, ng/mL	Week 0	54	29.7	22.8
	Week 8	33	51.4	44.0
	Week 16	33	90.6	79.5
TSAT^a^, %	Week 0	54	19.2	11.7
	Week 8	33	29.3	9.5
	Week 16	33	30.7	11.3
hs-CRP^b^, ng/mL	Week 0	54	2801.6	4134.1
	Week 16	32	5980.6	16,191.3

^a^Transferrin saturations

^b^High-sensitivity C-reactive protein

## Discussion and conclusion

In this study, hyperphosphatemia treatment with sucroferric oxyhydroxide (monotherapy or concomitant therapy with calcium carbonate) was found to successfully control serum phosphorus levels, even in HD patients who had received insufficient benefits from other phosphate-binders. In addition, target levels were achieved by Week 8 in 70% of patients, suggesting that this drug may have an early effect in terms of improvement in serum phosphorus levels. This result may reflect the fact that the equivalent dose of a sucroferric oxyhydroxide tablet is the highest of the commercially available phosphate-binders [20]. Calcium phosphate product is a possible factor affecting vascular calcification [21] and is recommended to be ≤ 55 mg^2^/dL^2^ [22]. In this study, calcium phosphate product levels decreased to within the target range in and after Week 8, which suggested that hyperphosphatemia treatment with sucroferric oxyhydroxide suppresses vascular calcification.

FGF23 may be more important than serum phosphorus, serum calcium, and PTH levels in the treatment of CKD-MBD [7]. Transcription of FGF23 has been reported to be enhanced by iron deficiency but not directly by serum phosphorus, serum calcium, or intact-PTH [23, 24], and an animal study reported that sucroferric oxyhydroxide, which contains iron, decreased FGF23 to a significantly greater extent than either lanthanum carbonate or sevelamer hydrochloride [17]. To the best of our knowledge, this study is the first clinical report indicating that the improvement in iron deficiency associated with hyperphosphatemia treatment with sucroferric oxyhydroxide possibly contributes directly to the decrease in FGF23 levels.

Increased Hb and decreased cumulative doses of ESAs and IV-iron were observed after administration of sucroferric oxyhydroxide, suggesting that the drug may act to improve renal anemia. These results may be attributed to the intestinal absorption of iron, a physiological metal contained in sucroferric oxyhydroxide [25]. Hb has been reported to affect vital prognosis [26], and overdoses of IV-iron have been reported to possibly increase cardiovascular risk and the risk of infection-related hospitalization [27–29]; therefore, administration of sucroferric oxyhydroxide may be expected to increase survival rate by improving renal anemia and avoiding the risks associated with IV-iron. Note that the mean of the cumulative dose of IV-iron had decreased significantly by Week 12 and had increased slightly by Week 16 in this study. This result can be attributed to the IV-iron that was administered for the comorbidity (subcutaneous hemorrhage) of pneumonia (1 patient), and no patient received IV-iron for renal anemia in Week 16.

Iron-based phosphorus binders are associated with a risk of iron overdose [30]. In this study, an increase in serum ferritin and TSAT level was observed, and the serum ferritin level continued to increase until Week 16. Although the means of these parameters were below 100 ng/mL, and were considered to remain at levels below those associated with safety concerns in Week 16, it may be necessary to monitor iron-related parameters during administration of sucroferric oxyhydroxide.

The most common adverse reaction in this study was diarrhea. Based on the results, sucroferric oxyhydroxide can be regarded as a drug that can safely control serum phosphorus levels when given as a 16-week treatment course. Non-compliance in HD patients affects overall mortality [31]. Although the benefits of sucroferric oxyhydroxide can be obtained with a smaller number of tablets than for sevelamer hydrochloride [11, 13], the tablets are relatively large, and in this study, administration was discontinued in four patients due to discomfort associated with study drug ingestion. To continue intake of sucroferric oxyhydroxide, a more sophisticated drug formulation are considered important.

The results of this study may be obtained not only by sucroferric oxyhydroxides but also through hyperphosphatemia treatment with sucroferric oxyhydroxide. There was also concern about the possibility that our results might be influenced by those patients who had been newly administered calcium carbonate after the start of the study (n = 6) and administered sucroferric oxyhydroxide in addition to calcium carbonate monotherapy (Adding group, n = 10). Therefore, we performed additional subgroup analyses on patients switched to sucroferric oxyhydroxide (Switching group, n = 24). As a result, the Switching group showed the same trends as full analysis set (n = 40). The findings from this study suggest that sucroferric oxyhydroxides reduce the disadvantages of existing hyperphosphatemia drugs and are useful therapeutic options for treating hyperphosphatemia in HD patients. Further studies involving more patients are needed.

In conclusion, the results of this study suggest that hyperphosphatemia treatment with sucroferric oxyhydroxide improves renal anemia and various factors affecting the vital prognosis of HD patients (such as FGF23, serum phosphorus, calcium phosphate product, and Hb levels and the use of IV-iron), and further investigation is expected.

## Limitations

This study is biased due to its design: a one-arm, open-label, before-and-after study. Intact-PTH was evaluated in only a few patients because it was measured only in patients who received cinacalcet hydrochloride tablets.

## Additional files

**Additional file 1: Additional methods.** Additional study design and statistical analysis.

**Additional file 2: Figure S1.** Patient disposition. A total of 54 patients were enrolled in this study, all of whom received sucroferric oxyhydroxide and were included in the safety analysis set (the population consisting of study subjects who received sucroferric oxyhydroxide at least once). Among these patients, 40 completed the observations in Week 8 and were included in the efficacy analysis set (the population consisting of patients who were continuing sucroferric oxyhydroxide treatment in Week 8 and have at least 1 analyzable data point for the efficacy endpoints in and after Week 8). Six patients were newly administered calcium carbonate after the start of the study. Ten patients were administered sucroferric oxyhydroxide in addition to calcium carbonate monotherapy (Adding group). Therefore, 24 patients were switched from existing hyperphosphatemia agents to sucroferric oxyhydroxide (Switching group). After the start of the study, 21 patients discontinued, and 33 patients completed the observations in Week 16.

**Additional file 3: Table S1.** Patient demographics and clinical characteristics (Switching group, n = 24).

**Additional file 4:Figure S2.** Time point at which target serum phosphorus level was achieved (All, n = 40). The breakdown of the first time of achieving the target serum phosphorus level (≥ 3.5 and ≤ 6 mg/dL) in the 40 patients of the efficacy analysis set.

**Additional file 5: Table S2.** Efficacy parameters of the Switching group and the Adding group (CKD-MBD parameters).

**Additional file 6: Table S3.** Efficacy parameters of the Switching group and the Adding group (renal anemia parameter).

**Additional file 7: Table S4.** Efficacy parameters of the Switching group and the Adding group (drug administration status).

**Additional file 8: Figure S3.** Time point at which target serum phosphorus level was achieved (Switching group, n = 24). The breakdown of the first time of achieving the target serum phosphorus level (≥ 3.5 and ≤ 6 mg/dL) in the 24 patients (Switching group).

**Additional file 9: Table S5.** **.** Adverse reactions occurred in 18 of 54 patients (33.3%); the most frequent event was diarrhea, which was observed in 9 of 54 patients (16.7%).

**Additional file 10: Table S6.** Safety parameters of the Switching group and the Adding group.

**Additional file 11: Table S7.** Adverse reactions of the Switching group and the Adding group.

## Abbreviations

CKD-MBD: chronic kidney disease-mineral and bone disorder
FGF23: fibroblast growth factor 23
HD: hemodialysis
ESAs: erythropoiesis-stimulating agents
IV-iron: intravenous iron
Hb: hemoglobin
hs-CRP: high-sensitivity C-reactive protein
SD: standard deviation
PTH: parathyroid hormone
TSAT: transferrin saturation
