# Supplementary material for: Sucroferric oxyhydroxide decreases serum phosphorus level and fibroblast growth factor 23 and improves renal anemia in hemodialysis patients
Source: BMC Res Notes. 2018 Jun 8;11:363. doi: 10.1186/s13104-018-3483-6 (PMC5994086; doi:10.1186/s13104-018-3483-6)
Supplement: Supplementary file 1 — Additional file 1: Additional methods. Additional study design and statistical analysis. [file 13104_2018_3483_MOESM1_ESM.pdf]

Supplementary methods

Study design

To achieve a target serum phosphorus level of ≥3.5 and ≤6.0 mg/dL, the dose of sucroferric oxyhydroxide was decreased or increased based on the patient's serum phosphorus level and any gastrointestinal symptoms. Administration of calcium carbonate was permitted only in patients who were already receiving such treatment prior to the start of the study, with its dose dependent upon serum levels of calcium and phosphorus. The concomitant use of oral iron supplements, peritoneal dialysis, and blood transfusions (except for transfusion of blood components other than red blood cell products) were prohibited during the study. Doses of erythropoiesis-stimulating agents (ESAs) and intravenous iron (IV-iron) were adjusted to achieve a target haemoglobin (Hb) level of ≥10 and <12 g/dL; changes in the type of ESA were not permitted during the study.

Statistical Analysis

Due to feasibility considerations for this exploratory study, the planned sample size was set at 50 patients. Efficacy endpoints were obtained from the full analysis set (FAS);

mean and standard deviation (SD) were calculated for the actual values and changes from baseline, and compared with baseline using the paired t-test. The following items were calculated at each evaluation time point after Week 8: the number of patients who had achieved or not achieved target serum phosphorus levels, the number of patients achieving the target for the first time at each time point, and the proportions of such within the analysis set. The analysis of safety endpoints was performed in the safety analysis set (SS), and the mean and SD of the actual values were calculated. For adverse events and adverse reactions, the number of patients, the number of cases, and the frequency were calculated. All statistical analyses were performed using SAS 9.4 software (SAS Institute Inc., Cary, NC, USA).
