# Supplementary material for: Sucroferric oxyhydroxide decreases serum phosphorus level and fibroblast growth factor 23 and improves renal anemia in hemodialysis patients
Source: BMC Res Notes. 2018 Jun 8;11:363. doi: 10.1186/s13104-018-3483-6 (PMC5994086; doi:10.1186/s13104-018-3483-6)
Supplement: Supplementary file 2 — Additional file 2: Figure S1. Patient disposition. A total of 54 patients were enrolled in this study, all of whom received sucroferric oxyhydroxide and were included in the safety analysis set (the population consisting of study subjects who received sucroferric oxyhydroxide at least once). Among these patients, 40 completed the observations in Week 8 and were included in the efficacy analysis set (the population consisting of patients who were continuing sucroferric oxyhydroxide treatment in Week 8 and have at least 1 analyzable data point for the efficacy endpoints in and after Week 8). Six patients were newly administered calcium carbonate after the start of the study. Ten patients were administered sucroferric oxyhydroxide in addition to calcium carbonate monotherapy (Adding group). Therefore, 24 patients were switched from existing hyperphosphatemia agents to sucroferric oxyhydroxide (Switching group). After the start of the study, 21 patients discontinued, and 33 patients completed the observations in Week 16. [file 13104_2018_3483_MOESM2_ESM.pdf]

Figure S1

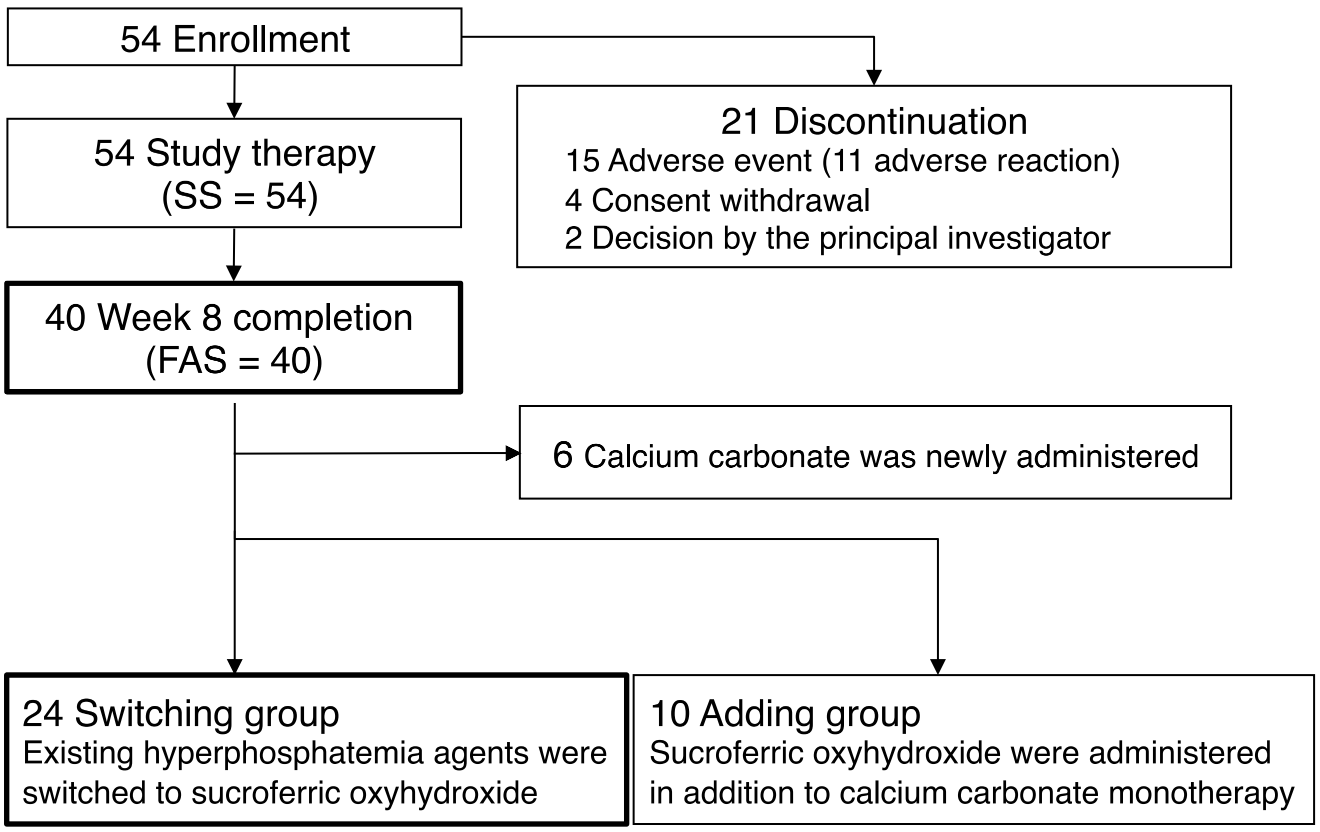

Figure S1. Patient disposition

A total of 54 patients were enrolled in this study, all of whom received sucroferric oxyhydroxide and were included in the safety analysis set (the population consisting of study subjects who received sucroferric oxyhydroxide at least once). Among these patients, 40 completed the observations in Week 8 and were included in the efficacy analysis set (the population consisting of patients who were continuing sucroferric oxyhydroxide treatment in Week 8 and have at least 1 analyzable data point for the efficacy endpoints in and after Week 8). Six patients were newly administered calcium carbonate after the start of the study. Ten patients were administered sucroferric oxyhydroxide in addition to calcium carbonate monotherapy (Adding group). Therefore, 24 patients were switched from existing hyperphosphatemia agents to sucroferric oxyhydroxide (Switching group). After the start of the study, 21 patients discontinued, and 33 patients completed the observations in Week 16.
