# Supplementary material for: Sucroferric oxyhydroxide decreases serum phosphorus level and fibroblast growth factor 23 and improves renal anemia in hemodialysis patients
Source: BMC Res Notes. 2018 Jun 8;11:363. doi: 10.1186/s13104-018-3483-6 (PMC5994086; doi:10.1186/s13104-018-3483-6)
Supplement: Supplementary file 3 — Additional file 3: Table S1. Patient demographics and clinical characteristics (Switching group, n = 24). [file 13104_2018_3483_MOESM3_ESM.pdf]

Table S1

Patient demographics and clinical characteristics (Switching group, n = 24)

Variable		n/mean	Percentage/ SD
Sex	Male	15	62.5
Age, year		64.5	12.7
Age	<65 years	8	33.3
	≥65 years	16	66.7
Dialysis method	Hemodialysis	8	33.3
	Hemodiafiltration	16	66.7
Dialysis history	<1 year	1	4.2
	≥1 and <3 years	4	16.7
	≥3 years	19	79.2
Primary disease of dialysis	Chronic glomerulonephritis[a]	11	45.8
	Diabetic nephropathy	5	20.8
	Nephrosclerosis	5	20.8
	Polycystic kidney	1	4.2
	Unknown	2	8.3
Complication	Hypertension	19	79.2
	Diabetes mellitus	8	33.3
	Dyslipidaemia	7	29.2
Prior hyperphosphatemia drug	Calcium carbonate	0	0.0
	Calcium carbonate + Lanthanum carbonate hydrate	7	29.2
	Calcium carbonate + Sevelamer hydrochloride	3	12.5
	Calcium carbonate + Bixalomer	3	12.5
	Calcium carbonate + Iron (III) citrate hydrate	2	8.3
	Calcium carbonate + Lanthanum carbonate hydrate + Iron (III) citrate hydrate	1	4.2
	Lanthanum carbonate hydrate	5	20.8
	Lanthanum carbonate hydrate + Iron (III) citrate hydrate	1	4.2
	Bixalomer	2	8.3
Daily dose of lanthanum carbonate hydrate, mg		1446.4	529.7
ESA[c] administration		23	95.8
Cumulative dose of ESAs[b], IU[c]		24782.6	17835.0
Intravenous iron administration		5	20.8
Cumulative dose of intravenous iron, mg		160.0	0.0

The values are mean (standard deviation) or n (%). Data for the 4 weeks before the start of the study shown for ESAs and intravenous iron. a: Including IgA nephropathy, b: erythropoiesis-stimulating agents, c: international unit.
