# Supplementary material for: Sucroferric oxyhydroxide decreases serum phosphorus level and fibroblast growth factor 23 and improves renal anemia in hemodialysis patients
Source: BMC Res Notes. 2018 Jun 8;11:363. doi: 10.1186/s13104-018-3483-6 (PMC5994086; doi:10.1186/s13104-018-3483-6)
Supplement: Supplementary file 4 — Additional file 4:Figure S2. Time point at which target serum phosphorus level was achieved (All, n = 40). The breakdown of the first time of achieving the target serum phosphorus level (≥ 3.5 and ≤ 6 mg/dL) in the 40 patients of the efficacy analysis set. [file 13104_2018_3483_MOESM4_ESM.pdf]

Figure S2

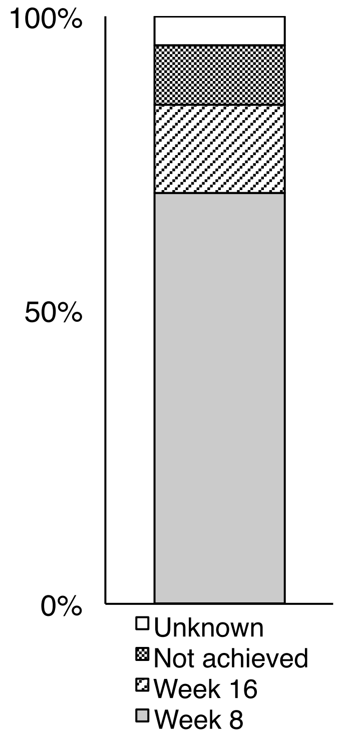

Figure S2. Time point at which target serum phosphorus level was achieved (All, n = 40)

The breakdown of the first time of achieving the target serum phosphorus level (≥3.5 and ≤6 mg/dL) in the 40 patients of the efficacy analysis set.
