# Supplementary material for: Sucroferric oxyhydroxide decreases serum phosphorus level and fibroblast growth factor 23 and improves renal anemia in hemodialysis patients
Source: BMC Res Notes. 2018 Jun 8;11:363. doi: 10.1186/s13104-018-3483-6 (PMC5994086; doi:10.1186/s13104-018-3483-6)
Supplement: Supplementary file 5 — Additional file 5: Table S2. Efficacy parameters of the Switching group and the Adding group (CKD-MBD parameters). [file 13104_2018_3483_MOESM5_ESM.pdf]

Table S2

Efficacy parameters of the Switching group and the Adding group (CKD-MBD parameters)

			Actual value			Changes			
			n	mean	SD	n	mean	SD	p-value[c]
Serum phosphorus, mg/dL	Baseline	Switching	24	7.0	0.7	-	-	-	-
		Adding	10	6.9	1.0	-	-	-	-
	Week 8	Switching	24	5.5	1.1	24	-1.5	1.3	<.0001
		Adding	9	5.2	1.6	9	-1.7	2.1	0.0399
	Week 16	Switching	19	5.6	1.4	19	-1.4	1.7	0.0017
		Adding	8	6.3	1.9	8	-0.9	2.1	0.2692
Corrected serum calcium, mg/dL	Baseline	Switching	24	8.7	1.0	-	-	-	-
		Adding	10	8.8	0.8	-	-	-	-
	Week 8	Switching	24	8.6	0.8	24	0.0	0.8	0.8291
		Adding	9	8.7	0.7	9	-0.1	0.9	0.8040
	Week 16	Switching	19	8.7	0.6	19	0.1	0.8	0.7460
		Adding	8	8.3	0.6	8	-0.3	0.8	0.3805
Calcium-phosphorus products, mg^2/dL^2	Baseline	Switching	24	60.4	9.1	-	-	-	-
		Adding	10	60.7	8.2	-	-	-	-
	Week 8	Switching	24	47.0	9.0	24	-13.4	11.6	<.0001
		Adding	9	44.8	11.2	9	-16.1	15.9	0.0158
	Week 16	Switching	19	48.8	12.5	19	-12.2	17.9	0.0084
		Adding	8	52.0	13.8	8	-9.8	17.2	0.1527
Serum intact-PTH[a], pg/mL	Baseline	Switching	16	210.9	136.6	-	-	-	-
		Adding	4	178.3	140.3	-	-	-	-
	Week 8	Switching	13	167.5	104.2	13	-12.5	82.6	0.5962
		Adding	1	73.0	-	1	-201.0	-	-
	Week 16	Switching	14	390.3	731.8	12	14.7	63.3	0.4393
		Adding	3	227.3	233.7	1	-172.0	-	-
FGF23[b], pg/mL	Baseline	Switching	24	17574.6	15321.5	-	-	-	-
		Adding	10	8920.0	7700.8	-	-	-	-
	Week 16	Switching	19	13027.9	12244.4	19	-4750.5	14260.4	0.1637
		Adding	8	4895.0	2946.0	8	-4230.0	7281.0	0.1443

a: parathyroid hormone, b: fibroblast growth factor 23, c: paired t-test (vs. baseline).
