# Supplementary material for: Sucroferric oxyhydroxide decreases serum phosphorus level and fibroblast growth factor 23 and improves renal anemia in hemodialysis patients
Source: BMC Res Notes. 2018 Jun 8;11:363. doi: 10.1186/s13104-018-3483-6 (PMC5994086; doi:10.1186/s13104-018-3483-6)
Supplement: Supplementary file 6 — Additional file 6: Table S3. Efficacy parameters of the Switching group and the Adding group (renal anemia parameter). [file 13104_2018_3483_MOESM6_ESM.pdf]

Table S3

Efficacy parameters of the Switching group and the Adding group (renal anemia parameter)

			Actual value			Changes			
			n	mean	SD	n	mean	SD	p-value[k]
Hb[a] g/dL	Baseline	Switching	24	11.1	1.1	-	-	-	-
		Adding	10	10.9	1.3	-	-	-	-
	Week 8	Switching	24	12.7	1.2	24	1.5	1.6	<.0001
		Adding	9	12.4	0.8	9	1.3	1.7	0.0581
	Week 16	Switching	19	12.5	1.1	19	1.4	1.5	0.0005
		Adding	8	13.2	1.8	8	2.7	2.8	0.0309
RBC[b], $\times 10^{-4}/\mu L$	Baseline	Switching	24	390.5	45.0	-	-	-	-
		Adding	10	397.0	20.6	-	-	-	-
	Week 8	Switching	24	418.2	46.3	24	27.7	38.2	0.0017
		Adding	9	419.2	47.4	9	21.9	40.3	0.1422
	Week 16	Switching	19	399.7	39.4	19	12.3	38.9	0.1847
		Adding	8	446.9	78.9	8	51.6	71.5	0.0803
Ht[c], %	Baseline	Switching	24	35.0	3.2	-	-	-	-
		Adding	10	34.4	3.1	-	-	-	-
	Week 8	Switching	24	39.4	4.0	24	4.4	4.5	<.0001
		Adding	9	38.7	3.1	9	3.8	5.2	0.0641
	Week 16	Switching	19	38.3	3.2	19	3.6	4.1	0.0013
		Adding	8	41.0	5.9	8	7.4	8.3	0.0400
Cumulative dose of ESAs[d], IU	Baseline[f]	Switching	24	23750.0	18161.7	-	-	-	-
		Adding	10	28000.0	22251.1	-	-	-	-
	Week 4[g]	Switching	22	24704.5	21305.4	22	-250.0	13688.5	0.9325
		Adding	10	25950.0	22879.5	10	-2050.0	11987.1	0.6018
	Week 8[h]	Switching	22	25534.1	18319.6	22	579.5	7752.6	0.7294
		Adding	9	35333.3	31020.2	9	6000.0	11478.2	0.1555
	Week 12[i]	Switching	24	13156.3	10381.8	24	-10593.8	16991.8	0.0056
		Adding	9	26777.8	24061.3	9	-2555.6	8560.2	0.3966
	Week 16[j]	Switching	20	11925.0	12004.4	20	-12075.0	23438.6	0.0327
		Adding	8	29000.0	18142.3	8	-1250.0	19001.9	0.8577
Cumulative dose of IV-iron[e], mg	Baseline[f]	Switching	24	33.3	66.4	-	-	-	-
		Adding	10	0.0	0.0	-	-	-	-
	Week 4[g]	Switching	22	18.2	38.5	22	-18.2	38.5	0.0379
		Adding	10	0.0	0.0	10	0.0	0.0	-
	Week 8[h]	Switching	22	0.0	0.0	22	-36.4	68.6	0.0215
		Adding	9	0.0	0.0	9	0.0	0.0	-
	Week 12[i]	Switching	24	0.0	0.0	24	-33.3	66.4	0.0218
		Adding	9	0.0	0.0	9	0.0	0.0	-
	Week 16[j]	Switching	20	0.0	0.0	20	-32.0	65.7	0.0421
		Adding	8	45.0	127.3	8	45.0	127.3	0.3506

a: hemoglobin, b: red blood cell count, c: hematocrit, d: erythropoiesis-stimulating agents, e: intravenous iron, f: Weeks -4 to 0, g: Weeks 0 to 4, h: Weeks 4 to 8, i: Weeks 8 to 12, j: Weeks 12 to 16, k: paired t-test (vs. baseline). Cumulative doses of ESAs and intravenous iron were regarded as 0 when they were not administered.
