# Supplementary material for: Sucroferric oxyhydroxide decreases serum phosphorus level and fibroblast growth factor 23 and improves renal anemia in hemodialysis patients
Source: BMC Res Notes. 2018 Jun 8;11:363. doi: 10.1186/s13104-018-3483-6 (PMC5994086; doi:10.1186/s13104-018-3483-6)
Supplement: Supplementary file 7 — Additional file 7: Table S4. Efficacy parameters of the Switching group and the Adding group (drug administration status). [file 13104_2018_3483_MOESM7_ESM.pdf]

Table S4

Efficacy parameters of the Switching group and the Adding group (drug administration status)

			Actual value			Changes			
			n	mean	SD	n	mean	SD	p-value[a]
Daily dose of calcium carbonate, g	Baseline	Switching	24	1.4	1.3	-	-	-	-
		Adding	10	1.9	0.8	-	-	-	-
	Week 4	Switching	22	1.4	1.4	22	0.1	0.3	0.3287
		Adding	10	1.8	0.7	10	-0.2	0.5	0.3434
	Week 8	Switching	24	1.5	1.4	24	0.1	0.4	0.1617
		Adding	9	1.8	0.7	9	-0.2	0.5	0.3466
	Week 12	Switching	24	1.4	1.4	24	0.0	0.5	0.7036
		Adding	10	1.5	0.7	10	-0.5	1.0	0.1934
	Week 16	Switching	19	1.4	1.5	19	0.0	0.7	1.0000
		Adding	8	1.4	0.8	8	-0.4	0.7	0.1705
Daily dose of sucroferric oxyhydroxide, mg	Baseline	Switching	24	1125.0	541.7	-	-	-	-
		Adding	10	700.0	105.4	-	-	-	-
	Week 4	Switching	22	1420.5	638.0	22	261.4	614.7	0.0593
		Adding	10	675.0	120.8	10	-25.0	79.1	0.3434
	Week 8	Switching	24	1458.3	632.7	24	333.3	606.4	0.0130
		Adding	9	694.4	110.2	9	-27.8	83.3	0.3466
	Week 12	Switching	24	1437.5	745.5	24	312.5	700.3	0.0393
		Adding	10	675.0	391.8	10	-25.0	342.6	0.8227
	Week 16	Switching	20	1487.5	784.2	20	325.0	702.9	0.0526
		Adding	8	812.5	458.1	8	93.8	441.9	0.5674

a: paired t-test (vs. baseline).
