# Supplementary material for: Sucroferric oxyhydroxide decreases serum phosphorus level and fibroblast growth factor 23 and improves renal anemia in hemodialysis patients
Source: BMC Res Notes. 2018 Jun 8;11:363. doi: 10.1186/s13104-018-3483-6 (PMC5994086; doi:10.1186/s13104-018-3483-6)
Supplement: Supplementary file 8 — Additional file 8: Figure S3. Time point at which target serum phosphorus level was achieved (Switching group, n = 24). The breakdown of the first time of achieving the target serum phosphorus level (≥ 3.5 and ≤ 6 mg/dL) in the 24 patients (Switching group). [file 13104_2018_3483_MOESM8_ESM.pdf]

Figure S3

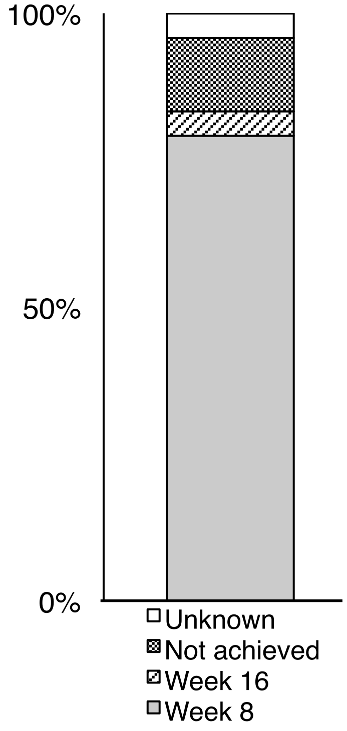

Figure S3. Time point at which target serum phosphorus level was achieved (Switching group, n=24)
The breakdown of the first time of achieving the target serum phosphorus level (≥3.5 and ≤6 mg/dL) in the 24 patients (Switching group).
