# Supplementary material for: Sucroferric oxyhydroxide decreases serum phosphorus level and fibroblast growth factor 23 and improves renal anemia in hemodialysis patients
Source: BMC Res Notes. 2018 Jun 8;11:363. doi: 10.1186/s13104-018-3483-6 (PMC5994086; doi:10.1186/s13104-018-3483-6)
Supplement: Supplementary file 9 — Additional file 9: Table S5. . Adverse reactions occurred in 18 of 54 patients (33.3%); the most frequent event was diarrhea, which was observed in 9 of 54 patients (16.7%). [file 13104_2018_3483_MOESM9_ESM.pdf]

Table S5

Adverse reactions (All, n = 54)

	cases	patients	%
Adverse reaction	21	18	33.3
Diarrhea	9	9	16.7
Constipation	3	3	5.6
Abdominal pain	1	1	1.9
Abdominal discomfort	2	2	3.7
Hemoglobin increased	4	4	7.4
Others	2	2	3.7
