# Supplementary material for: Sucroferric oxyhydroxide decreases serum phosphorus level and fibroblast growth factor 23 and improves renal anemia in hemodialysis patients
Source: BMC Res Notes. 2018 Jun 8;11:363. doi: 10.1186/s13104-018-3483-6 (PMC5994086; doi:10.1186/s13104-018-3483-6)
Supplement: Supplementary file 10 — Additional file 10: Table S6. Safety parameters of the Switching group and the Adding group. [file 13104_2018_3483_MOESM10_ESM.pdf]

Table S6

Safety parameters of the Switching group and the Adding group

			Actual value		
			n	mean	SD
Serum iron, µg/dL	Week 0	Switching	35	62.8	35.0
		Adding	13	54.2	31.8
	Week 8	Switching	20	89.2	23.0
		Adding	8	62.1	19.8
	Week 16	Switching	19	80.7	21.9
		Adding	8	54.4	23.9
Serum ferritin, ng/mL	Week 0	Switching	35	32.0	24.0
		Adding	13	27.4	23.8
	Week 8	Switching	20	47.5	23.6
		Adding	8	63.8	81.7
	Week 16	Switching	19	104.6	90.8
		Adding	8	49.9	22.2
TSAT[a], %	Week 0	Switching	35	20.7	12.2
		Adding	13	16.4	10.4
	Week 8	Switching	20	32.0	9.7
		Adding	8	22.5	8.1
	Week 16	Switching	19	31.6	8.7
		Adding	8	20.1	9.1
hs-CRP[b], ng/mL	Week 0	Switching	35	2127.4	2611.5
		Adding	13	4807.8	6833.6
	Week 16	Switching	18	3752.1	6165.0
		Adding	8	14832.4	30719.1

a: transferrin saturations, b: high-sensitivity C-reactive protein
