# Supplementary material for: Sucroferric oxyhydroxide decreases serum phosphorus level and fibroblast growth factor 23 and improves renal anemia in hemodialysis patients
Source: BMC Res Notes. 2018 Jun 8;11:363. doi: 10.1186/s13104-018-3483-6 (PMC5994086; doi:10.1186/s13104-018-3483-6)
Supplement: Supplementary file 11 — Additional file 11: Table S7. Adverse reactions of the Switching group and the Adding group. [file 13104_2018_3483_MOESM11_ESM.pdf]

Table S7

Adverse reactions of the Switching group and the Adding group

	Switching group (n = 35)			Adding group (n = 13)		
	cases	patients	%	cases	patients	%
Adverse reaction	15	13	37.1	4	3	23.1
Diarrhea	6	6	17.1	3	3	23.1
Constipation	2	2	5.7	1	1	7.7
Abdominal pain	1	1	2.9	0	0	0.0
Abdominal discomfort	1	1	2.9	0	0	0.0
Hemoglobin increased	3	3	8.6	0	0	0.0
Others	2	2	5.7	0	0	0.0
